# A tile model of circuit topology for self-entangled biopolymers

**DOI:** 10.1038/s41598-023-35771-8

**Published:** 2023-06-01

**Authors:** Erica Flapan, Alireza Mashaghi, Helen Wong

**Affiliations:** 1 Mathematics and Statistics Department, Pomona College, Claremont, CA 91711 USA; 2 Faculty of Science, Leiden University, 2333CC Leiden, The Netherlands; 3 Mathematical Sciences Department, Claremont McKenna College, Claremont, CA 91711 USA

**Keywords:** Applied mathematics, Molecular biology, Protein folding

## Abstract

Building on the theory of circuit topology for intra-chain contacts in entangled proteins, we introduce tiles as a way to rigorously model local entanglements which are held in place by molecular forces. We develop operations that combine tiles so that entangled chains can be represented by algebraic expressions. Then we use our model to show that the only knot types that such entangled chains can have are $3_1$, $4_1$, $5_1$, $5_2$, $6_1$, $6_2$, $6_3$, $7_7$, $8_{12}$ and connected sums of these knots. This includes all proteins knots that have thus far been identified.

## Introduction

Entanglement is believed to affect the healthy function of proteins and can play a role in misfolding diseases including neurodegeneration, muscular dystrophy, and some forms of cancer, yet a rigorous characterization of entanglement in proteins remains elusive^1–4^. It has been established that knots, slipknots and other complex topologies exist in cellular proteins^5–9^ and have been conserved throughout evolution^10–12^. While knots and links in protein backbones have been characterized^5,7,9^, and links and lassos involving intra-chain bonds have been identified^5,6^, studying each of these features in isolation provides only a limited view of the topological entanglement of a molecule. What is needed is a comprehensive analysis of entanglement that includes the intertwining of the backbone and intra-chain bonds (e.g. disulfide bridges or hydrogen bonds) as well as the tangling of sites which may be distant on the amino acid sequence but are twisted together and held in place by molecular forces.

**Figure 1:**
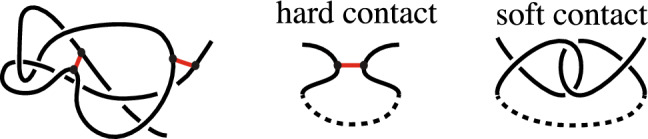
Left: intertwining of the backbone and the intra-chain bonds, known as hard contacts, shown in red; right: two distant sites that are tangled together through the formation of a soft contact.

The framework of circuit topology was introduced by Mashaghi^13,14^ as one approach to developing such a comprehensive analysis. Circuit topology describes the simplest fold units of biopolymers consisting of intra-chain contacts (known as hard contacts) and locally entangled units which use molecular forces to hold segments of the chain together (known as soft contacts). For example, in Fig. 1, the red segments in the left image represent hard contacts, while the intertwined arcs in the right image represent a soft contact. Four distinct contacts are recognized in the original circuit topology framework^15^ as the most basic units of entanglement.

Entangled linear biopolymers such as proteins can then be described in terms of hard and soft contacts together with operations which combine them^13,15–17^. There is data indicating that the folding kinetics of polymer chains is correlated with the number and arrangement of hard contacts, and circuit topology operations for hard contacts can be used to describe the protein folding and evolution^18–22^. The success of the circuit topology framework for hard contacts together with the potential biological significance of soft contacts have motivated us to seek a rigorous approach to soft contacts. Such a theory may also facilitate molecular engineering, and thus find use in a wide range of applications, including nanotechnology and soft matter design^15,23,24^.

The aim of this paper is to model soft contacts in a single entangled biopolymer chain by using specific projections of 1-string and 2-string tangles based on entangled fold units observed in Golovnev et al.^14^. Since these basic units are held in place by molecular forces, we treat their projections as rigid objects and represent them as rectangles which we refer to as tiles. We obtain tile complexes by joining tiles according to circuit topology operations introduced by Mashaghi. We define operation notation in order to represent tile complexes symbolically according to how they were constructed. Next we show that the only knots which can be obtained from tile complexes by joining their endpoints are $3_1$, $4_1$, $5_1$, $5_2$, $6_1$, $6_2$, $6_3$, $7_7$, $8_{12}$ together with connected sums of these knots. This includes all known protein knots^7,9^. We introduce sequence notation to represent entanglements whose construction pathway are unknown. Finally, we present an algorithm to go from any sequence that satisfies two simple requirements to a picture of its tile complex together with operation notation describing how it might have been constructed.

## Tiles

### Definition 1.1

A rectangle in the plane with compass points is one where the short sides are labeled E (East) and W (West), the long sides are labeled N (North) and S (South), and the NE, NW, SW, SE points occur at the Northeast, Northwest, Southwest, and Southeast corners, respectively. A 1-string tile is a projection of a 1-string tangle on such a rectangle oriented from one endpoint on the E side to the other endpoint on the W side. A 2-string tile is a projection of a 2-string tangle on such a rectangle with one endpoint in each of the corners.

**Figure 2:**
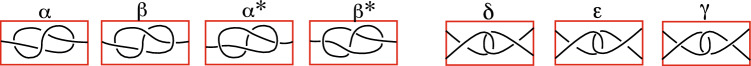
These tiles are based on the projections in^14^.

Note that if compass points are not specified for a horizontal tile, we assume they are in the standard positions. In Fig. 2, we introduce four 1-string tiles and three 2-string tiles based on the projections of locally entangled proteins illustrated in Fig. 8A of Golovnev et al.^14^ and the bottom of Fig. 3 of Golovnev et al.^14^. Since the two 1-string tiles on the right are obtained from those on the left by reflecting through the plane of the paper, we label them with an $^*$, as is typically done for the mirror image of knots.

We will use the tiles in Fig. 2 as building blocks, and obtain additional tiles by rigid motions of these tiles.

### Definition 1.2

Tiles *A* and *B* are said to be equal, denoted $A= B$, if there is a rigid planar motion from *A* to *B* such that the compass points NE, NW, SW, SE of *A* go to the compass points NE, NW, SW, SE, of *B* respectively. If there is no such motion, then we say *A* and *B* are distinct and write $A \not = B$.

### Definition 1.3

Let *A* be a tile. The long axis that goes across *A* is the *x*-axis, the short axis that goes across *A* is the *y*-axis , and the line through the center point of *A* perpendicular to the plane of the paper is the *z*-axis. These axes determine the *yz*-, *xz*-, and *xy*-planes associated with *A*. We use the following notation.

- $A_x$ is the result of rotating *A* by $180^\circ$ around its *x*-axis.
- $A_y$ is the result of rotating *A* by $180^\circ$ around its *y*-axis.
- $A_z$ is the result of rotating *A* by $180^\circ$ around its *z*-axis.
- $A_v$ is the result of reflecting *A* across its *yz*-plane.
- $A_h$ is the result of reflecting *A* across its *xz*-plane.
- $A^*$ is the result of reflecting *A* across its *xy*-plane.

**Figure 3:**
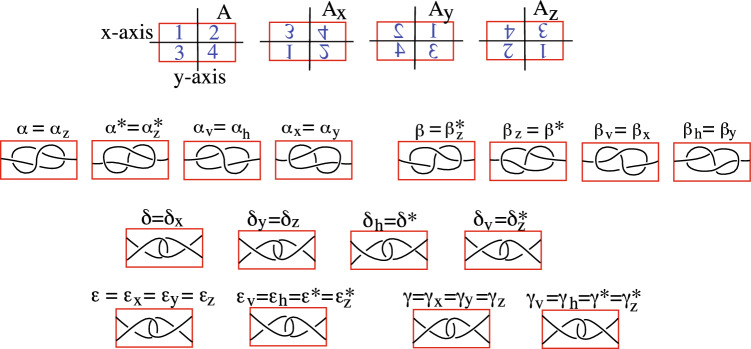
Top: rotations of a rectangle around *x*-axis, *y*-axis, and *z*-axis. Middle: tiles obtained from $\alpha$ and $\beta$ by doing rotations and reflections. Bottom: tiles obtained from $\delta$, $\varepsilon$, and $\gamma$ by doing rotations and reflections.

The results of rotating a rectangle around the *x*-, *y*-, and *z*-axes are illustrated in the top image of Fig. 3. If we compose two of these rotations, we obtain the third rotation. Thus these are the only $180^\circ$ rotations of a tile. If we compose two reflections, we obtain a rotation. Finally, if we compose a *z*-rotation and a reflection across the *xy*-plane we obtain an inversion. Rather than introducing an additional notation for an inversion we denote it as the composition $A_z^*$.

The following propositions tell us all of the tiles we can obtain by rotating or reflecting $\alpha$, $\beta$, $\delta$, $\varepsilon$, and $\gamma$. The proofs follow from the middle and bottom images in Fig. 3.

### Proposition 1.4

$\alpha$ *and* $\beta$ *together with their rotations and reflections give us the following eight distinct tiles*.

- $$\alpha =\alpha _z$$, $$\alpha ^*=\alpha _z^*$$, $$\alpha _v = \alpha _h$$, $$\alpha _x = \alpha _y$$.
- $$\beta =\beta _z^*$$, $$\beta _z = \beta ^*$$, $$\beta _v = \beta _x$$, $$\beta _h = \beta _y$$.

### Proposition 1.5

$\delta$, $\varepsilon$, *and* $\gamma$ *together with their rotations and reflections give us the following eight distinct tiles*.

- $$\delta =\delta _x$$, $$\delta _y = \delta _z$$, $$\delta _h = \delta ^*$$, $$\delta _v=\delta _z^*$$.
- $$\varepsilon = \varepsilon _x = \varepsilon _y = \varepsilon _z$$, $$\varepsilon ^* = \varepsilon _v = \varepsilon _h=\varepsilon _z^*$$.
- $$\gamma = \gamma _x = \gamma _y = \gamma _z$$, $$\gamma ^* = \gamma _v = \gamma _h=\gamma _z^*$$.

The 16 tiles in the middle and bottom images of Fig. 3 are the only ones that we allow.

## Operations and tile complexes

**Figure 4:**
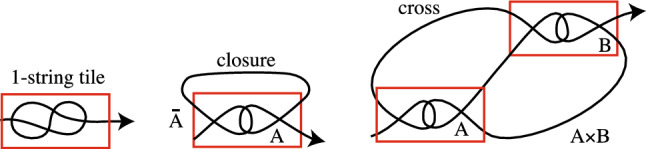
These are the basic tile complexes .

Next, we will join tiles together with arcs in the plane to obtain a projection of a single entangled oriented arc that we refer to as a tile complex. This entangled arc represents a single protein or other polymer chain. All 1-string tangles are tile complexes where they are oriented from their W endpoint to their E endpoint. In the left image in Fig. 4, we add an arrow to a 1-string tile to indicate the orientation. Note that in what follows, we omit over-under information and use Roman rather than Greek letters so that our pictures can represent any 1- or 2-string tile.

### Definition 2.1

The closure of a 2-string tile *A*, denoted by $\overline{A}$, is obtained by joining the NE and NW endpoints of *A* with a planar arc disjoint from *A* to obtain a single arc which is oriented from its W endpoint to its E endpoint. See the middle image in Fig. 4.

### Definition 2.2

The cross operation for 2-string tiles *A* and *B*, denoted $A\times B$, is obtained by adding arcs joining the NW endpoint of *A* to the NE endpoint of *A, the SE endpoint of A* to the SE endpoint of *B*, and the NE endpoint of *A* to the SW endpoint of *B*, such that the new arcs are disjoint from *A*, *B*, and each other. We orient $A\times B$ from the endpoint on the W side of *A* to the endpoint on the E side of *B*. See the right image in Fig. 4.

The three basic tile complexes are illustrated in Fig. 4. These definitions guarantee that all of the crossings in a basic tile complex are contained in the tiles and that its endpoints will be in the same region of the plane. This latter requirement is important in order to be able to join the endpoints of a tile complex together in the plane without introducing any new crossings. We now introduce operations which allow us to combine tile complexes to obtain more complicated tile complexes.

### Definition 2.3

The series operation $S+T$ is obtained by adding a planar arc *p* joining the E endpoint of a tile complex *S* to the W endpoint of a tile complex *T*, such that the interior of *p* is disjoint from *S* and *T*. $S+T$ is oriented from the W endpoint of *S* to the E endpoint of *T*.

### Definition 2.4

The parallel operation $S\parallel T$ inserts a tile complex *T* into an interior arc *p* of a tile complex *S*, such that *p* is not contained in a tile and the orientation on *S* agrees with that on *T*. $S\parallel T$ is oriented from the W endpoint of *S* to the E endpoint of *S*.

**Figure 5:**
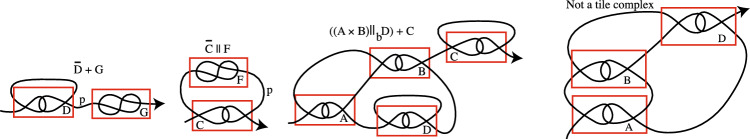
Some examples of tile complexes and an entangled arc which is not a tile complex.

On the left of Fig. 5, we have added an arc *p* joining the W endpoint of the 2-string tile closure $\overline{D}$ to the E endpoint of the 1-string tile *G*, while in the next image we have inserted the 1-string tile *F* into an arc *p* of the 2-string tile closure $\overline{C}$. In the third image, we illustrate an example with multiple operations. The image on the right is not a tile complex because it does not correspond to any of our operations.

More generally, when we insert a tile complex into $A\times B$, we use the subscripts *b* (bottom), *m* (middle), or *t* (top) to indicate which arc of $A\times B$ we are inserting into; and when we are inserting into $S\parallel T$, we use the subscript *E* (East) and *W* (West) to indicate which arc of $S\parallel T$ we are inserting into.

### Definition 2.5

A projection *K* of an entangled arc is said to be realizable as a tile complex *T* if there is an isotopy of space taking *T* to *K* treating each tile as a rigid object.

In the Supplemental Material, we prove that no matter how we join the two ends of a 2-string tile to itself to create an arc (as in the closure), join two 2-string tiles to obtain an arc that alternates between the two tiles (as in the cross), join two tile complexes together with an arc (as in series), or insert a tile complex into an interior arc of another tile complex not contained in a tile such that it respects orientation (as in parallel), we will obtain the same set of entangled arcs which are realizable as tile complexes. Thus the choices we have made in our definitions are not important in terms of the types of entangled arcs that we obtain as tile complexes.

### Definition 2.6

We say tile complexes S and T are equal and write $S = T$ if there is a planar isotopy taking the tiles and arcs of *S* to the tiles and arcs of *T*, respectively, treating the tiles as rigid so that the W and E endpoints of *S* go to the W and E endpoints of *T* respectively.

Note that it is possible for tile complexes to be equal even though they are constructed differently and have different notation. For example, $(\overline{A}\parallel \overline{C})\parallel _W\overline{B}=(\overline{A}\parallel \overline{B})\parallel _E\overline{C}=\overline{A}\parallel (\overline{B}+\overline{C})$.

## Sealing tile complexes

We would like to model knotted proteins by tile complexes, then determine all possible knot types that can occur. Since all of the crossings of a tile complex are within its tiles which are treated as rigid objects, the tangling of a tile complex is trapped in the tile complex. This is in contrast with when we model knotted proteins as topological arcs in space where the tangling can fall off of the ends if we do not pin them down or join them. While this is not an issue with tile complexes, we want to join the ends together in order to obtain a knot, which we will consider up to isotopy in space.

By the definition of our operations, the endpoints of a tile complex are always in the same region of the plane. Thus we can join them in the plane without introducing any additional crossings. Up to a planar isotopy, there is only one way to join the endpoints of a tile complex by a planar arc which is disjoint from the complex. This means that the following definition is unambiguous.

### Definition 3.1

Let *S* and *T* be tile complexes.The sealing of *S* is the knot *K*(*S*) whose projection is obtained by joining the endpoints of *S* via a planar arc disjoint from *S*. We write $K(S)\thicksim K(T)$ and say that *K*(*S*) and *K*(*T*) have the same knot type if they are isotopic as knots in 3-dimensional space.

**Figure 6:**
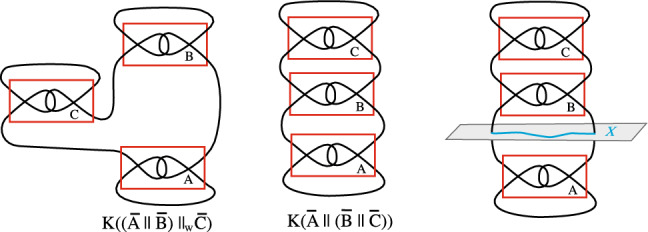
Left and center: the sealings $K((\overline{A}\parallel \overline{B})\parallel _W\overline{C})\thicksim K(\overline{A}\parallel (\overline{B}\parallel \overline{C}))$, but $(\overline{A}\parallel \overline{B})\parallel _W\overline{C}\ne \overline{A}\parallel (\overline{B}\parallel \overline{C})$. Right: $K(\overline{A})\#(K(\overline{B})\#K(\overline{C}))$.

In contrast with tile complexes, when we consider sealings, we allow deformations of the arcs in space both inside and outside of the tiles. Because of this, tile complexes whose sealings have the same knot type are not necessarily equal as tile complexes. For example, we see on the left and center in Fig. 6 that the sealings $K((\overline{A}\parallel \overline{B})\parallel _W\overline{C})$ and $K(\overline{A}\parallel (\overline{B}\parallel \overline{C}))$ are isotopic since the knotted arc $\overline{C}$ in $K((\overline{A}\parallel \overline{B})\parallel _W\overline{C})$ can be slid along an arc of $\overline{B}$ to place it at the top of $\overline{B}$. However, as tile complexes $(\overline{A}\parallel \overline{B})\parallel _W\overline{C}$ and $\overline{A}\parallel (\overline{B}\parallel \overline{C})$ are not equal.

### Definition 3.2

We say that a knot *K* is the connected sum of knots $K_1$ and $K_2$, and write $K\thicksim K_1\#K_2$, if there is a topological plane *P* in space that meets *K* in just two points and an arc *X* in *P* whose endpoints coincide with the points $P\cap K$ such that if we join *X* to one component of $K-P$ we get $K_1$ and if we join *X* to the other component of $K-P$ we get $K_2$.

For example, the knot in Fig. 6 is the connected sum $K(\overline{A})\#(K(\overline{B})\#K(\overline{C}))$ illustrated on the right. In fact, the connected sum operation is associative so we do not need the parentheses around $K(\overline{B})\#K(\overline{C})$. The observations below follow from the definitions of the connected sum and the series and parallel operations.

### Obsertion 3.3

Let *S* and *T* be tile complexes. Then

1. $$K(S + T)\thicksim K(S) \#K(T)$$.
2. $$K(S \parallel T) \thicksim K(S) \# K(T)$$.

The following lemma reduces our analysis of knot types to determining the knot types of the sealings of the three basic tile complexes.

### Lemma 3.4

*The sealing of every tile complex is a connected sum (possibly with only one non-trivial summand), where each non-trivial summand comes from the sealing of a basic tile complex*.

### *Proof*

Recall that a basic tile complex is either a 1-string tile, the closure of a 2-string tile, or the cross of two 2-string tiles. By definition, every tile complex is constructed from basic tile complexes by repeatedly applying the series and/or parallel operations. Thus we can repeatedly apply Observation 3.3 to conclude that the sealing of any tile complex is a connected sum as required. $\square$

The following lemma, proved in the Supplemental Material, determines the knot type of the sealing of the basic tile complexes.

### Lemma 3.5

*If* *A* *is a* 1-*string tile, then* *K*(*A*) *is the trefoil knot* $\pm 3_1$ *or the Figure-eight knot* $4_1$. *If* *B* *is a* 2-*string tile, then* $K(\overline{B})$ *is* $0_1$ (*i.e., the trivial knot*) , $\pm 3_1$, *or* $4_1$. *If* *A* *and* *B* *are* 2-*string tiles, then* each of *A* *and* *B* is *isotopic in space fixing its endpoints to one of* $\delta$, $\delta ^*$, $\varepsilon$, $\varepsilon ^*$, $\gamma$, $\gamma^*$, *and* $K(A \times B)$ *or its mirror image is* *Table* 1.

**Table 1:** All knot types that can be obtained as the cross of two 2-string tiles.

Cross of 2-string tiles	Knot type	Cross of 2-string tiles	knot type
$K(\delta \times \delta )$	$3_1$	$K(\delta ^* \times \delta )$	$4_1$
$K(\delta \times \varepsilon )$	$5_2$	$K(\delta ^* \times \varepsilon )$	$0_1$
$K(\delta \times \gamma )$	$6_1$	$K(\delta ^* \times \gamma )$	$0_1$
$K(\varepsilon \times \varepsilon )$	$5_1$	$K(\varepsilon ^* \times \varepsilon )$	$6_3$
$K(\varepsilon \times \gamma )$	$6_2$	$K(\varepsilon ^* \times \gamma )$	$7_6$
$K(\gamma \times \gamma )$	$7_7$	$K(\gamma ^* \times \gamma )$	$8_{12}$

### Theorem 3.6

*The sealing of a tile complex has the knot type of a connected sum of the knots* $0_1$, $3_1$, $4_1$, $5_1$, $5_2$, $6_1$, $6_2$, $6_3$, $7_6$, $7_7$, $8_{12}$ *or their mirror images*.

### *Proof*

By Lemma 3.5, the sealing of a basic tile complex is $0_1$, $3_1$, $4_1$, $5_1$, $5_2$, $6_1$, $6_2$, $6_3$, $7_6$, $7_7$, $8_{12}$ or the mirror image of one of these. By Lemma 3.4, every tile complex is a connected sum of the sealings of basic tile complexes. $\square$

## Sequence notation

We saw above that we can represent a tile complex by an algebraic expression that describes how we constructed it. Such an expression will be referred to as operation notation for the tile complex. This notation is convenient for keeping track of how the tile complex was constructed, however it has its limitations. A given tile complex could be constructed in more than one way and hence have more than one operation notation. For example, we noted previously that $(\overline{A}\parallel \overline{C})\parallel _W\overline{B}=(\overline{A}\parallel \overline{B})\parallel _E\overline{C}=\overline{A}\parallel (\overline{B}+\overline{C})$. Also, if a collection of tiles and arcs joining them is not a tile complex, it will not have operation notation. For example, the image on the right of Fig. 5 illustrates a configuration of tiles and arcs which does not correspond to a tile complex because the cross operation is only defined for two 2-string tiles. In a future paper, we consider tile complexes where the definition of the cross operation is extended to stacks of 2-string tiles, as on the right in Fig. 5.

We now introduce a different notation for tile complexes, which ignores how a tile complex is constructed, but can be easily read off from a picture of tiles and arcs joining them even if the picture is not a tile complex. We will call this notation sequence notation. In contrast with operation notation, a given projection of an entangled arc as a tile complex always has unique sequence notation.

Given a tile complex, we define its sequence notation as the consecutive list of letters representing the tiles (where every tile has a unique letter) as we travel along the oriented arc going from its W endpoint to its E endpoint. This is well defined for every tile complex, since every tile complex has well defined W and E endpoints. On the other hand, not every sequence of letters corresponds to a tile complex. For example, the sequence *ABDBAD* (whose configuration is illustrated on the right in Fig. 5) might seem to correspond to $(\overline{A}\parallel \overline{B})\times D$, but we can only take the cross of a pair of 2-string tiles so this sequence does not represent a tile complex. In order to avoid sequences which do not represent tile complexes, we only consider sequences that satisfy the following requirements.

**Requirements for sequences to represent tile complexes**

1. Each letter either appears once and represents a 1-string tile or appears twice and represents a 2-string tile.
2. At most one letter can alternate with a given letter, and any letters which alternate must represent 2-string tiles.

Observe that the sequence *ABDBAD* violates Requirement (2) because *D* alternates with *A* and *B*. Thus this sequence is excluded.

We introduce the following terminology to refer to particular strings of letters in a sequence. If a letter appears only once in a sequence we call it a singleton and by Requirement 1 it represents a 1-string tile. If a letter appears twice in a sequence then together they represent a 2-string tile. We refer to consecutive instances of the same letter such as *AA* as twins, and adjacent alternating letters such as *ABAB* as an interweaving. The former represents $\overline{A}$ and the latter represents $A\times B$. Note that alternating pairs of letters *A* and *B* which are not adjacent in a sequence, such as *ABACCB*, represent a cross of *A* and *B* with additional tile complexes inserted, but such a sequence is not considered to be an interweaving since the last *B* is not adjacent to the last *A*.

Using the following lemma (proved in the Supplemental Material), we will give an algorithm to go from a sequence satisfying our two requirements to a drawing of a tile complex and operation notation representing how the tile complex could be constructed. There is an example given after the algorithm.

### Lemma 4.1

*Any sequence with no singletons which satisfies Requirements 1 and 2 must either have a pair of twins or an interweaving.*

**Algorithm to go from a sequence to a tile complex with operation notation**

**Step 0.** Let $S_0$ denote a sequence satisfying the two Requirements.

**Step 1.** Let $S_1$ denote the sequence obtained by deleting the singletons from $S_0$. If $S_0$ has no singletons, then let $S_1=S_0$. Observe that $S_1$ is the empty sequence precisely when all of the letters in $S_0$ were singletons. In this case, go to Step 8.

**Step 2.** Let $k=1$. Since $S_k$ is non-empty and has no singletons, it follows from Lemma 4.1 that $S_k$ either has a pair of twins or an interweaving. Let $S_{k+1}$ denote the sequence obtained by deleting the pairs of twins and interweavings from $S_k$.

**Step 3.** Repeat Step 2 with $k=2$, 3, ...to go from each sequence $S_{k}$ to a shorter sequence $S_{k+1}$. Stop when we obtain the empty sequence $S_n$.

**Step 4.** Draw $S_{n-1}$ as a line oriented from W to E, with each of its pairs of twins and interweavings marked such that if a pair of twins or interweaving is to the left of another in the sequence $S_{n-1}$, then its position is to the left of the other on the line.

**Step 5.** For each marking representing a pair of twins or an interweaving on the line draw the closure of the appropriate 2-string tile or the cross of the appropriate 2-string tiles, respectively. This gives us the tile complex with sequence $S_{n-1}$. Now let $i=n-1$.

Operation notation: The operation notation for $S_i$ is a series where each summand is of the form $\overline{A}$ for each pair of twins and of the form $A \times B$ for each interweaving, with the summands in the order in which they occurred on the line.

**Step 6.** For any pair of twins or interweavings from $S_{i-1}$ which were deleted in $S_{i}$, we put closures or crosses, respectively, into the tile complex for $S_i$ in the appropriate places to get a tile complex for $S_{i-1}$.

Operation notation: Use the series or parallel operations to place these closures and crosses in the appropriate places of the operation notation for $S_i$, which gives us the operation notation for $S_{i-1}$. Note that there may be a subscript on $\parallel$ to indicate the exact position of the insertion. Also, use parentheses as necessary to resolve any ambiguities.

**Step 7.** Repeat Step 6, for each $i<n-1$ until we get a tile complex with operation notation for $S_1$.

**Step 8.** Now insert any singletons that were in $S_0$ into appropriate arcs of the tile complex for $S_1$.

Operation notation: use series or parallel to place these 1-string tiles in the appropriate places in the operation notation for $S_1$ to get the operation notation for $S_0$. $\Box$

**Figure 7:**
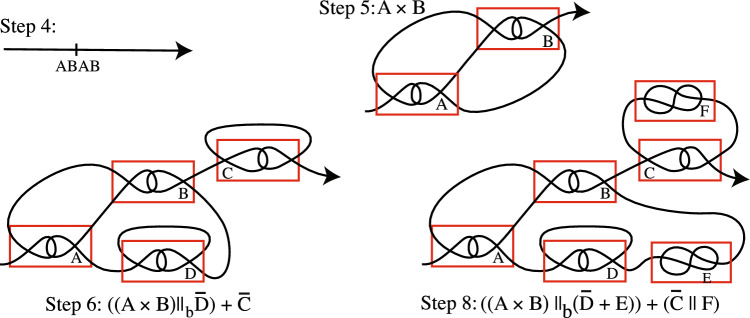
We apply our algorithm to $S_0=ABADDEBCFC$ to get a tile complex with operation notation $((A\times B)\parallel _{b} (\overline{D}+E))+ (\overline{C}\parallel F)$.

In order to illustrate our algorithm, below we apply it to the sequence *ABADDEBCFC* to obtain a tile complex together with its operation notation. Figure 7 illustrates Steps 4–8 for this example.

**Step 0:** Let $$S_0=ABADDEBCFC$$.

**Step 1:** Delete the singletons *E* and *F* from $S_0$ to obtain $S_1=ABADDBCC$.

**Step 2:** Delete *DD* and *CC* from $S_1$ to get $S_2=ABAB$.

**Step 3:** Delete the interweaving *ABAB* to get the empty sequence $S_3$.

**Step 4:** Draw a line oriented from W to E to represent $S_2$ and mark *ABAB* on it.

**Step 5:** Replace *ABAB* on the line by a drawing of $A\times B$ to get the tile complex for $S_2$.

**Step 6:** Since $S_1=ABADDBCC$ contains the twins *DD* and *CC*, insert $\overline{D}$ into the bottom string of $A\times B$ and put $\overline{C}$ on the right end of the string. This is the tile complex for $S_1$ with operation notation $((A\times B)\parallel _b\overline{D})+\overline{C}$.

**Step 7:** Since we have a configuration for $S_1$, we do not repeat Step 6.

**Step 8:** We insert the 1-string tile *E* to the right of $\overline{D}$, and insert the 1-string tile *F* in the arc at the top of $\overline{C}$. This is a drawing of $S_0=ABADDEBCFC$ which has operation notation $((A\times B)\parallel _b(\overline{D}+E))+(\overline{C}\parallel F)$.

Observe that there is only one way to follow the steps of our algorithm to go from a sequence to a tile complex and operation notation, and a given tile complex has only one sequence. However, there may be multiple operation notations which give us the same tile complex and sequence. For example, the tile complex $(\overline{A}\parallel \overline{C})\parallel _W\overline{B}=(\overline{A}\parallel \overline{B})\parallel _E\overline{C}=\overline{A}\parallel (\overline{B}+\overline{C})$ has the sequence *ABBCCA*. But if we apply our algorithm to the sequence *ABBCCA*, we only get the operation notation $\overline{A}\parallel (\overline{B}+\overline{C})$. So while our algorithm determines an operation notation for a given sequence, the sequence and its tile complex might also be represented by other operation notations.

## Discussion

Chemists have long speculated that the 3D architecture of biopolymers is as crucial in determining their physiological properties as the chemical structure of their monomers^25^. It is now known that the central dogma in molecular biology, which states that the amino acid sequence (i.e., 1D arrangement of amino acids) of a protein should be sufficient to describe its structure and function, fails in many cases^26,27^. For this and other reasons, there has been extensive research aimed at finding descriptors of 3D conformation^28,29^. In this regard, one approach that appeared to be fruitful was describing the 3D structure in terms of elementary building blocks. One such elementary building block is seen in the secondary structures of proteins, namely alpha helices and beta sheets. Although secondary structure analysis has been useful in the case of stably folded proteins (even though sequence-independent interactions may contribute to secondary structure stability^30,31^), it has limited applicability in the case of intrinsically disordered proteins (IDPs) that largely lack such secondary structures^32^. Furthermore, alpha helices and beta structures are typically seen in polypeptides and related polymers, and they cannot be seen generically as building blocks of folded linear polymers of arbitrary chemistry. For example, single-chain nanoparticles made of various chemistries exhibit self-entanglements and complex folding^33^. To describe these self-entangled and folded conformations from the bottom up, the circuit topology framework was formulated^14,15^.

In this paper, we use the framework of circuit topology to present a rigorous mathematical theory of local entanglement in which tiles are building blocks that represent soft contacts. By starting with a fixed set of tiles and piecing them together via well defined operations, we are able to algebraically express and analyze the entanglement of a single linear (bio)polymer, such as a protein molecule. In addition to such operation notation, we introduce sequence notation to provide a rigorous way to measure the complexity of global entanglement. Both operation notation and sequence notation are robust descriptors, and we provide an algorithm to go from one to the other for any tile complex. Finally, we show that our tile model captures the topological knot types of all currently known protein knots^7,9^ as well as that of more complex protein knots that have yet to be identified. In future work, we extend our model to multiple (bio)polymer chains that are tangled together and to include intrachain bonds (known as hard contacts) as well as soft contacts.

Analogous to how mechanical and folding kinetic properties of folded polymers were shown to be correlated with the number and arrangement of hard contacts^18–20,34^, we expect that the same will be true for soft contacts, though this has yet to be verified experimentally. Our tile-based model provides rigorous definitions for how to count the number and measure the complexity of the arrangement of soft contacts, and thus makes it possible to design experiments to test the mechanical properties of complexes made from self-entangled building blocks.

Additionally, mechanical or thermal stress can cause a folded chain to undergo conformational change, which could result in the formation or disruption of contacts. For hard contacts, the dynamics have been mapped into a topological landscape by counting the number of hard contacts arranged in different topological manners^32,35^. While our tile model focuses on analyzing a single conformation of an entangled chain (a static picture), we can take a similar approach to dynamically track entanglement by sampling conformations over time and counting the number and complexity of the arrangement of soft contacts using our tile model.

Perhaps most importantly, we believe that our bottom-up approach contributes to the sequential synthesis of entangled structures and will thus be valuable to researchers interested in the emerging field of programmed polymer folding^23^. Materials made from knotted structures are widely used due to the longevity and inherent mechanical robustness that results from the intricate interplay of their topology, elasticity, and friction. Our tile model will enable the engineering of such materials with emergent mechanical properties by piecing together self-entangled building blocks in ways that are described mathematically as operations on tiles in our model. Combining our approach with soft matter physics efforts could thus lead to the design and development of complex, self-entangled structures that possess a wide range of mechanical properties^36,37^.

## Methods

We used techniques from knot theory to obtain all of the results in the paper. Detailed proofs of the independence of the definitions of the tile operations, and of Lemmas 3.5 and 4.1 are included in the Supplementary Information.

## Supplementary Information

Supplementary Information.

## Data Availability

All data is contained in the article and the Supporting Information.
