# Supplementary material for: A tile model of circuit topology for self-entangled biopolymers
Source: Sci Rep. 2023 Jun 1;13:8889. doi: 10.1038/s41598-023-35771-8 (PMC10235088; doi:10.1038/s41598-023-35771-8)
Supplement: Supplementary file 1 — Supplementary Information. [file 41598_2023_35771_MOESM1_ESM.pdf]

# Supporting Information to: A tile model of circuit topology for self-entangled biopolymers

Erica Flapan, Alireza Mashaghi, and Helen Wong*

E-mail: eflapan@pomona.edu

## Independence of how tile operations are defined

Recall the following definition from Section 3 of the paper.

**Definition 0.1** *A projection $K$ of an entangled arc is said to be* realizable as a tile complex *$T$ if there is an isotopy of 3-dimensional space taking $T$ to $K$, treating each tile as a rigid object.*

In the lemmas below, we prove that no matter how we define the tile operations, we will obtain entangled arcs which are realizable as the tile complexes defined in the paper. This means that defining the operations as we do in the paper does not limit the entanglements that we obtain.

**Lemma 0.2** *If any planar arc is added to a 2-string tile to obtain the projection of an arc whose endpoints are in the same region of the plane, the resulting arc is realizable as the closure of a 2-string tile.*

**Proof.** While we can connect two endpoints of a 2-string tile in multiple ways, if we connect two endpoints on a single short side of the rectangle this will give us a loop and an arc. If we connect two diagonal endpoints of a tile, it will isolate one endpoint of the arc from the other.

There are only two remaining ways to connect the ends of a 2-string tile to itself. Connecting the NW and NE endpoints of a 2-string tile $A$ yields the tile complex $\overline{A}$. Connecting the SW and SE endpoints of $A$ and then rotating around the $x$-axis of the tile yields the tile complex $\overline{A_x}$ (see Figure 1). Hence, both ways of connecting are realizable as a tile complex. □

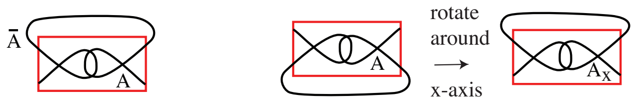


Figure 1: If we connect the SW and SE endpoints of $A$ we obtain an entanglement that is realizable as $\overline{A}_x$.

By analogy with the cross operation introduced in Golonev and Mashaghi,[1] the cross of two 2-string tiles should be an arc which alternates between the two tiles. We obtain this by joining the two tiles by three planar arcs such that the endpoints of the resulting arc are in the same region of the plane. However, as we see in Figure 2, there is more than one way to do this.

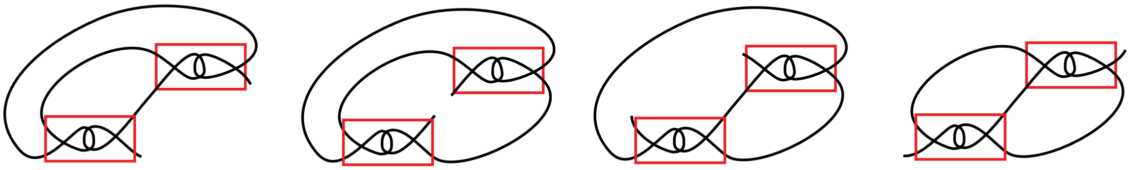

Figure 2: We illustrate four ways of adding three arcs between two 2-string tiles so that the endpoints of the resulting arc are in the same region of the plane.

**Lemma 0.3** *Figure 2 illustrates the only ways to add three arcs between two 2-string tiles so that the endpoints of the resulting arc are in the same region of the plane. Each of these configurations is realizable as the cross of two 2-string tiles.*

In order to prove this lemma, we introduce the following notation. The subscript $xy$ on a tiles indicates that the tile has first been rotated around the $x$-axis (i.e., the long axis) and then been rotated around the $y$-axis (i.e., the short axis). Together these give us a rotation around the $z$-axis (i.e., the central point). Similarly $yx$ denotes a rotation around the $y$-axis

followed by one around the $x$-axis, which is again just a rotation around the $z$-axis. So in fact, the order in which we do the rotations makes no difference.

**Proof.** To make our argument more symmetric, we draw one 2-string tile as horizontal and the other as vertical. In Figure 3, we illustrate the four ways to join the NW corner of a horizontal 2-string tile to a corner of a vertical 2-string tile. Observe that once a single arc is added, there is only one way to add arcs between the remaining pairs of corners so that the four arcs are pairwise disjoint. This can be done by joining adjacent pairs of corners going clockwise around one rectangle while going counterclockwise around the other rectangle.

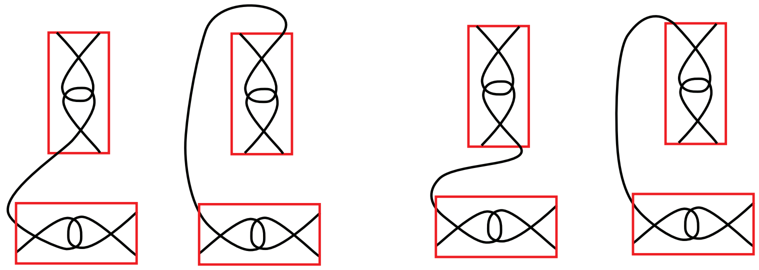

Figure 3: The four ways to join the NW corner of a horizontal 2-string tile to a corner of a vertical 2-string tile.

If we join adjacent corners of a 2-string tile along a short side to adjacent corners of another 2-string tile along a short side, we get a closed loop that only includes one string of each tile (see the left side of Figure 4). Thus adding two more arcs to the right two pictures

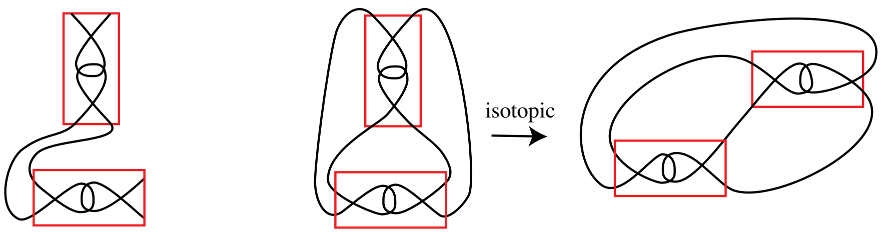


Figure 4: Left: Joining adjacent corners of a 2-string tile along a short side to adjacent corners of another 2-string tile along a short side gives a closed loop. Right: This is the only way to join the corners of two 2-string tiles with mutually disjoint arcs to obtain a single loop.

in Figure 3 would not lead to a single arc as required by the lemma. Also, there is a planar isotopy between the two left pictures in Figure 3. Thus, we can assume without loss of

generality that we have the first picture, and the only way to join the corners of the tiles with mutually disjoint arcs to get a single closed loop is illustrated in the middle image of Figure 4.

Note there is a planar isotopy which takes the middle picture of Figure 4 to the one on the right. Thus any set of three arcs between two 2-string tiles which yield a single arc whose endpoints are in the same region of the plane must be obtained by removing one of the four arcs between the tiles on the right in Figure 4. The four configurations in Figure 2 are the only ways to do so.

In order to see that all four configurations illustrated in Figure 2 are realizable as the cross of two tiles, first observe that there is a planar isotopy taking the first, second, third, and fourth pictures in Figure 2 to the first, second, third, and fourth pictures in Figure 5, respectively. Next observe that if we rotate the tiles around their $x$- or $y$-axis and turn the picture over as indicated we go from the first to the second to the third to the fourth configuration by an isotopy that treats the tiles as rigid. Since the fourth configuration is our definition of the cross operation, we conclude that these three configurations are all realizable as a cross of two 2-string tiles. □

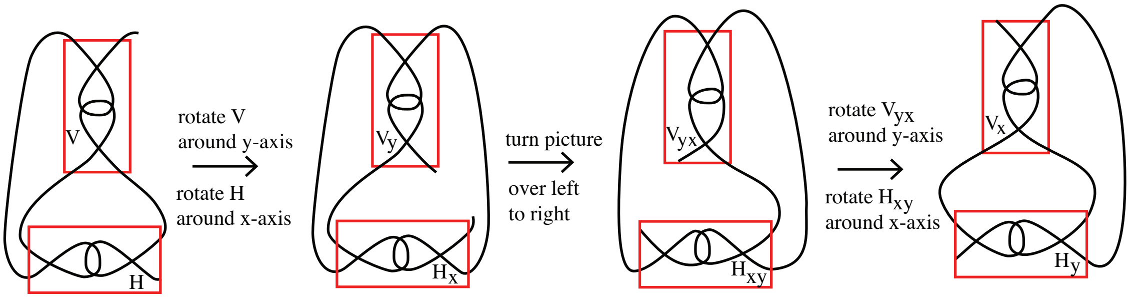


Figure 5: The configurations in Figure 2 are realizable as the cross of two 2-string tiles.

**Lemma 0.4** *No matter how two tile complexes are joined by a planar arc so that the resulting arc has a single orientation, we obtain the series of two tile complexes.*

**Proof.** Connecting the E endpoint of $S$ to the W endpoint of $T$ yields $S + T$. Connecting the W endpoint of $S$ to the E endpoint of $T$ yields $T + S$. If we connect the two E endpoints

or the two W endpoints, the resulting arc would not have a single orientation. □

**Lemma 0.5** *There is only one way for a tile complex $T$ to be inserted into an interior arc $p$ of a tile complex $S$ so that $p$ is disjoint from the tiles and the resulting arc has a single orientation. Moreover, the result yields $S \parallel T$.*

**Proof.** Note that $p$ inherits an orientation from $S$, and thus has a well-defined E endpoint and W endpoint. In order for the resulting arc to have a single orientation, the W endpoint of $T$ must be inserted into the W endpoint of $p$ and the E endpoint of $T$ into the E endpoint of $p$. This yields $S \parallel T$. □

Observe that we cannot slide a tile complex along the string inside of another tile complex to change the order of the tile complexes. Thus, if $S \neq T$, then $S + T \neq T + S$ and $S \parallel T \neq T \parallel S$.

# Verification of knot types of sealings of basic tile complexes

In the paper, we saw that the sealing of a tile complex is the connected sum of sealings of basic tile complexes, which are 1-string tiles, closures of 2-string tiles, and the cross of two 2-string tiles. In the lemmas below, we prove that the sealings of the basic tile complexes are $0_1$, $3_1$, $4_1$, $5_1$, $5_2$, $6_1$, $6_3$, $7_7$, $8_{12}$ or their mirror images.

**Lemma 0.6** *If $A$ is a 1-string tile, then $K(A)$ is the trefoil knot $\pm 3_1$ or the figure-eight knot $4_1$.*

**Proof.** In Figure 6, we draw the sealings of the 1-string tiles and see that all those arising from $\alpha$ are the $\pm 3_1$ knot and all arising from $\beta$ are the achiral knot $4_1$. □

**Lemma 0.7** *If $B$ is a 2-string tile, then $K(\overline{B})$ is $0_1$* (i.e., the trivial knot), *$\pm 3_1$, or $4_1$.*

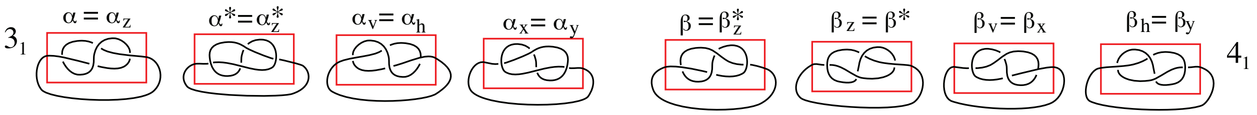


Figure 6: The sealings of the 1-string tiles with their knot types.

**Proof.** In Figure 7, we draw the sealings of the 2-string tiles and see that all those arising from $\delta$ are the trivial knot $0_1$, all those arising from $\varepsilon$ are the trefoil knot $\pm 3_1$, and all those arising from $\gamma$ are the figure eight knot $4_1$. $\square$

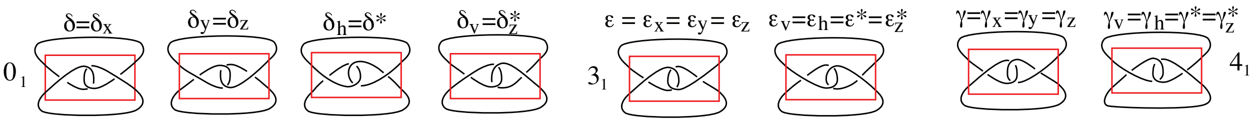


Figure 7: The sealings of the 2-string tiles with their knot types.

Before we determine the sealings of the cross of two 2-string tiles, we prove the following technical lemma.

**Lemma 0.8** *Let $A$ and $B$ be 2-string tiles.*

1. *Either $A$ is equal to $A_z$, or there is an isotopy of space taking $A$ to $A_z$ fixing the endpoints of the arcs. It follows that, up to an isotopy in space fixing the endpoints of the arcs, the only 2-string tiles are $\delta$, $\delta^*$, $\varepsilon$, $\varepsilon^*$, $\gamma$, $\gamma^*$*

2. *$K(A \times B) \sim K(B \times A)$.*

3. *$(A \times B)^* = A^* \times B^*$.*

**Proof.** (1) We saw in Proposition 2.5 of the paper that if $A$ is one of the 2-string tiles $\varepsilon$, $\varepsilon^*$, $\gamma$, and $\gamma^*$, then that $A = A_z$, and if $A$ is $\delta$, $\delta_y$, $\delta_v$ or $\delta_h$, then $A_z$ is $\delta_y$, $\delta$, $\delta_h$, or $\delta_v$, respectively. Figure 8 shows that $\delta$ and $\delta_y$ are related by an isotopy of the strings that turns the center part over by 360° and switches the outer crossings, while fixing the endpoints of the strings. Similarly, $\delta_h$ and $\delta_v$ are related by an isotopy that turns the center part by 360° in the opposite direction, fixing the endpoints of the strings. Thus the only 2-string tiles up to isotopy of space are $\delta$, $\delta^*$, $\varepsilon$, $\varepsilon^*$, $\gamma$, $\gamma^*$.

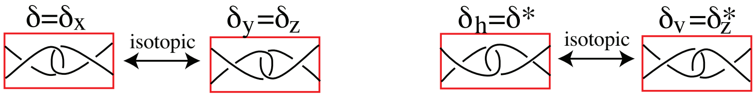


Figure 8: $\delta$ and $\delta_y$ are isotopic and $\delta_h$ and $\delta_v$ are isotopic by isotopies fixing the endpoints of the arcs.

(2) By Part (1), regardless of which 2-string tiles they represent, there are isotopies taking $A$ to $A_z$ and $B$ to $B_z$ fixing the endpoints of the arcs. Since the arcs connecting $A$ and $B$ in $A \times B$ are unaffected by such isotopies, these isotopies can be combined to create an isotopy from $K(B \times A)$ to $K(B_z \times A_z)$.

Next, we see in Figure 9 that $K(B_z \times A_z) \sim K(A \times B)$.

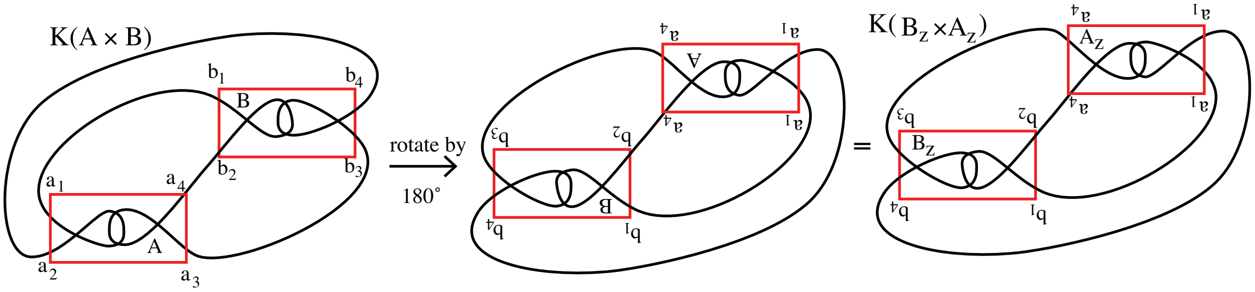


Figure 9: An isotopy in space takes $K(A \times B)$ to $K(B_z \times A_z)$.

(3) We take the mirror image by reflecting in the plane of the paper. All over-crossings become under-crossings, and all under-crossings become over-crossings. Since all the crossings in $A \times B$ are inside the tiles, it follows that $(A \times B)^* = A^* \times B^*$. □

**Lemma 0.9** *If $A$ and $B$ are 2-string tiles, then $K(A \times B)$ has knot type* $0_1$, $3_1$, $4_1$, $5_1$, $5_2$, $6_1$, $6_2$, $6_3$, $7_7$, $8_{12}$ *or their mirror images.*

**Proof.** We saw in Part (1) of Lemma 0.8 that we need only to consider the tiles $\delta$, $\delta^*$, $\varepsilon$, $\varepsilon^*$, $\gamma$, and $\gamma^*$ when we are determining knot types. By Lemma 0.8, we have $K(A \times B) \sim K(B \times A)$. Also, by Part (3), $K(A \times B)^* \sim K(A^* \times B^*)$. Hence, up to taking mirror images, there are twelve possibilities for the sealing of a cross of two 2-string tiles. We evaluate the knot type of each of these cases in the images below. □

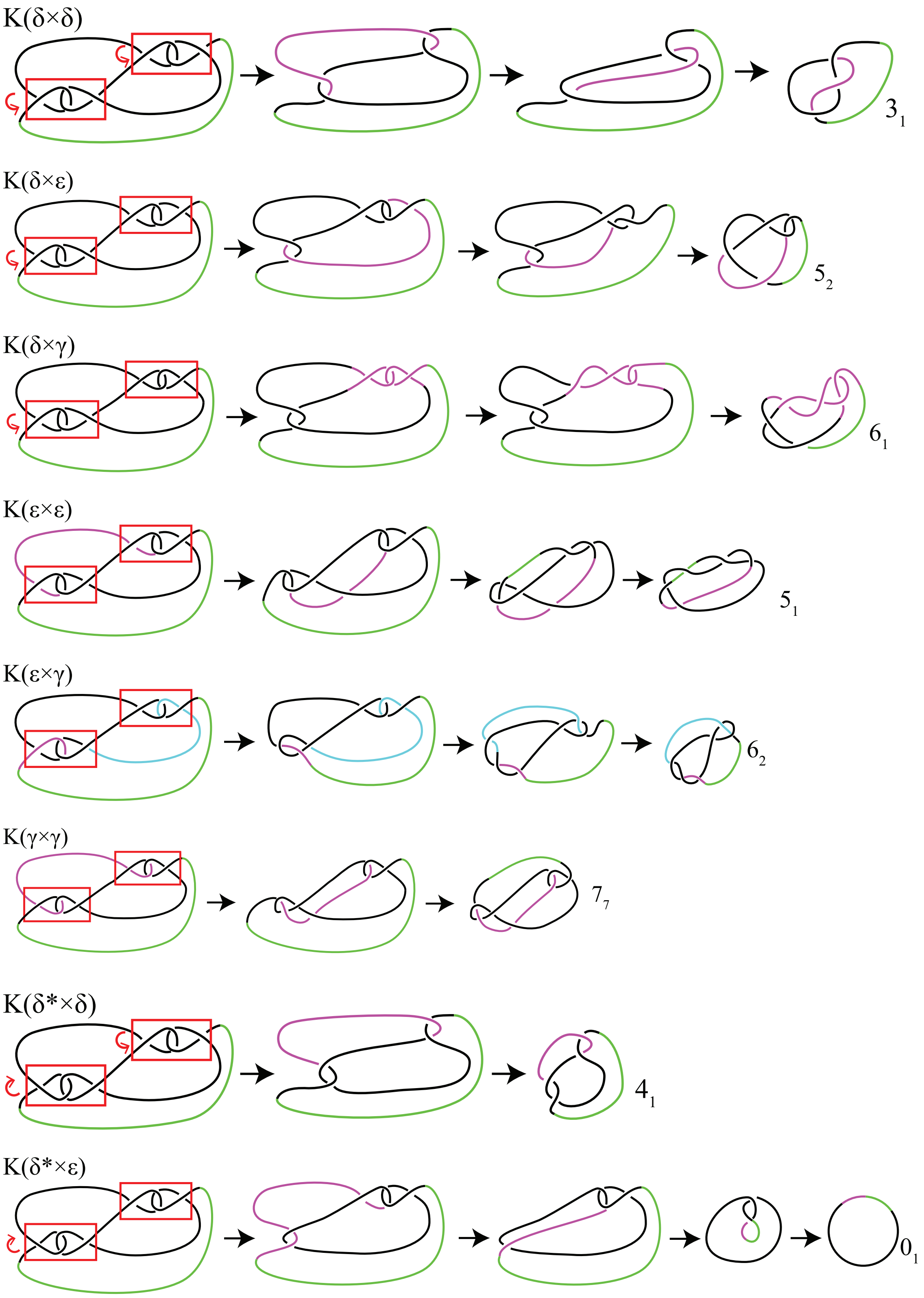
K(δ×δ)
3_1
K(δ×ε)
5_2
K(δ×γ)
6_1
K(ε×ε)
5_1
K(ε×γ)
6_2
K(γ×γ)
7_7
K(δ*×δ)
4_1
K(δ*×ε)
0_1

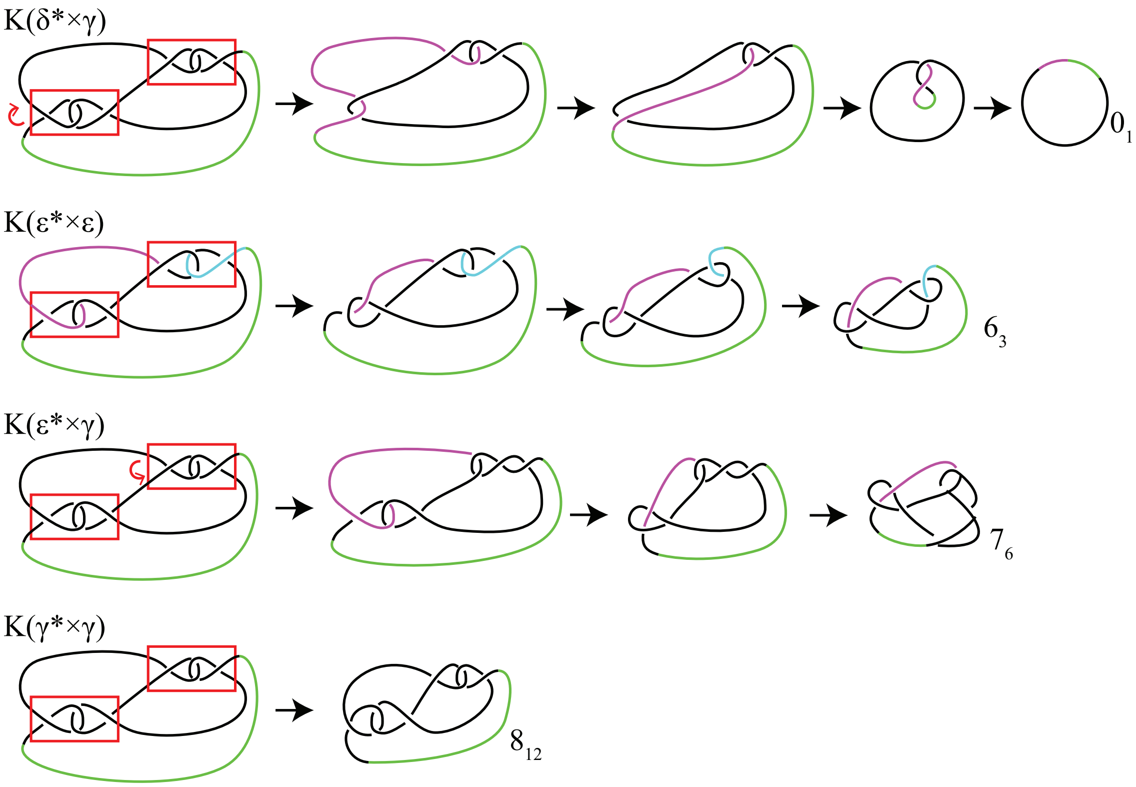


## Twins and interweavings in sequence notation

The algorithm to go from sequence notation to operation notation for a tile complex relies on a lemma that guarantees, in the absence of singletons, the existence of a pair of twins or an interweaving in the sequence. We provide its complete proof here. Recall the *distance* between two letters in a sequence is the number of letters from the first letter to the second letter. For example, in $ABACCB$ the distance from $A$ to its partner is 2 while the distance from $C$ to its partner is 1.

**Lemma 0.10 (Lemma 5.1 in main text)** *Any sequence with no singletons which satisfies Requirements 1 and 2 must either have a pair of twins or an interweaving.*

**Proof.** Suppose there is a sequence with no singletons, twins, or interweavings. Then every letter in the sequence has a partner and every pair of partners has at least one letter between them. Let $A$ be a letter whose distance from its partner is minimal among all letters in the

sequence, and let $B$ be a letter between the two $A$'s. Because the distance between the two $A$'s is minimal, no letter can occur twice between the two $A$'s. Thus $B$'s partner is not between the two $A$'s. By Requirement 2, at most one letter can alternate with $A$, and hence $B$ is the only letter between the two $A$'s. It follows that $ABA$ occurs within our sequence.

Now among all occurrences of the form $ABA$, choose one where the string $S$ of letters between $ABA$ and the other $B$ has minimal distance from its partner. Since the sequence has no interweavings, $S$ cannot be empty. However, because no letter besides $A$ can alternate with $B$, any letter in $S$ also has its partner in $S$. So we can choose $C$ in $S$ so that the distance from $C$ to its partner is minimal. Now apply the above paragraph to the sequence $S$ with $C$ in place of $A$, to obtain a $D$ such that $CDC$ occurs in $S$. Since both $D$'s must be in $S$, the number of letters between $CDC$ and the other $D$ is less than the length of $S$. But this contradicts the minimality of the length of $S$. Thus our assumption that there is a sequence with no singletons, twins, or interweavings was wrong. □

# References Cited


(1) Golovnev, A.; Mashaghi, A. Generalized circuit topology of folded linear chains. *iScience* **2020**, *23*, 101492.
